# CD80-CD28 signaling controls the progression of inflammatory colorectal carcinogenesis

**DOI:** 10.18632/oncotarget.2780

**Published:** 2015-01-16

**Authors:** Marco Scarpa, Paola Brun, Melania Scarpa, Susan Morgan, Andrea Porzionato, Andromachi Kotsafti, Marina Bortolami, Andrea Buda, Renata D'Incà, Veronica Macchi, Giacomo C. Sturniolo, Massimo Rugge, Romeo Bardini, Ignazio Castagliuolo, Imerio Angriman, Carlo Castoro

**Affiliations:** ^1^ Oncological Surgery Unit, Veneto Institute of Oncology IOV – IRCCS, Padova 35128, Italy; ^2^ Department of Molecular Medicine, University of Padova, Padova 35128, Italy; ^3^ Department of Histopathology, Sheffield Teaching Hospitals, Sheffield S10 2JF, UK; ^4^ Department of Surgery Oncology and Gastroenterology, University of Padova, Padova 35128, Italy; ^5^ Department of Medicine, University of Padova, Padova 35128, Italy

**Keywords:** ulcerative colitis, colorectal carcinogenesis, antigen presenting cells, CD8+ T cells, intestinal epithelial cells

## Abstract

In patients with ulcerative colitis (UC) the cumulative risk of colon cancer is lower than the actual rate of dysplasia suggesting an efficient immune surveillance mechanism. Since the co-stimulatory molecule CD80 is overexpressed in dysplastic colonic mucosa of UC patients and T-cell activation entails effective costimulation, we aimed to evaluate the functional implication of CD80 signaling in colonic UC-associated carcinogenesis. In humans, we observed that the percentage of CD80+ and HLA-A+ IEC was increased in the dysplastic colonic mucosa of UC patients. *In vitro*, IEC activated CD8+ T-cells through a CD80-dependent pathway. Finally, in the AOM/DSS-induced colonic adenocarcinoma model CD80 signaling inhibition significantly increased the frequency and extension of high-grade dysplasia, whereas enhancing CD80 activity with an anti-CTLA4 antibody significantly decreased colonic dysplasia. In conclusion, CD80 signaling between IEC and T-cells represents a key factor controlling the progression from low to high grade dysplasia in inflammatory colonic carcinogenesis.

## INTRODUCTION

Ulcerative colitis (UC) is a chronic inflammatory disorder involving the rectum and to a various extent the colon [1]. UC patients experience several complications including an increased risk of colorectal cancer (CRC) [1]. Several independent risk factors for malignancy in UC patients have been identified such as duration of disease, extent of inflammation, history of concurrent primary sclerosing cholangitis and family history of CRC [2]. Pre-malignant histological alterations in UC patients are broadly referred to as dysplasia, rather than adenoma, since dysplasia is frequently not polypoid [3, 4]. Moreover, several molecular pathways such as wnt-βcatenin pathway are involved in colonic carcinogenesis in UC [5]. Current guidelines for management of dysplasia in UC recommend total proctocolectomy in case of high grade dysplasia (HGD) or in case of two consecutive observation of low grade dysplasia (LGD) [6]. Intriguingly, the cumulative risk of colon cancer in patients with UC is approximately 8% twenty years after the diagnosis, although the cumulative rate of dysplasia is at least 25% [7–8]. Moreover, proctocolectomy specimens from UC patients with preoperative diagnosis of dysplasia exhibit neoplastic lesions in only two third of cases [9], and 64% of UC patients with LGD exhibit indefinite or no dysplasia after 4-years follow-up [10]. These inconsistencies show a fluid and variable presence of dysplastic lesions in the inflamed colonic mucosa indicating the possibility of a regression of preneoplastic lesions. These observations suggest the presence of efficient immune surveillance mechanisms that lead to neoplastic cells destruction (immune surveillance), possibly mediated by T-cells, macrophages, and natural killer cells [11].

Successful T-cell activation entails effective costimulation signaling mediated by CD80, CD86 or CD40 on the antigen-presenting cells (APC) and CD28 or CD40L receptors on T-cells [12–14]. CTLA4, a member of the immunoglobulin superfamily expressed on the T-cell surface, can compete with CD28 for binding to CD80 and CD86, transmitting an inhibitory signal to T cells instead [15]. CD80 expression can be induced by oncogenic insults [16], including oxidative DNA damage associated to intestinal chronic inflammation [17], suggesting a major role in immune surveillance mechanisms. In a previous study, we reported CD80 mRNA upregulation in UC preneoplastic lesions and its down-regulation in invasive cancer [17, 18]. However, the precise role of CD80 signaling and its regulation during UC-related colonic carcinogenesis remains unclear.

Several studies have investigated T-cell activation in CRC but few data are available concerning its role in the early stages of inflammatory carcinogenesis. A significantly higher proportion of activated CD8 T-cells expressing CD69 and CD107 in early invasive cancer compared to advanced cancer in non inflammatory conditions has been described [19]. Pages et al., meanwhile, showed that non inflammatory CRC without signs of early metastatic invasion have increased infiltrates of immune cells and higher levels of downstream products of Th1 activation but not of inflammatory or immunosuppressive mediators [20]. However, a clear characterization of the immune microenvironment, and in particular of T cells activation, in colonic inflammatory carcinogenesis is still lacking. Therefore, the aim of our study was to evaluate the functional implication of CD80 signaling in the epithelial-CD8+T-cells cross-talk in immune surveillance mechanisms during UC-associated carcinogenesis of the colon.

## RESULTS

### Human epithelial cells can act as antigen presenting cells in UC-related colonic carcinogenesis

**Figure 1:**
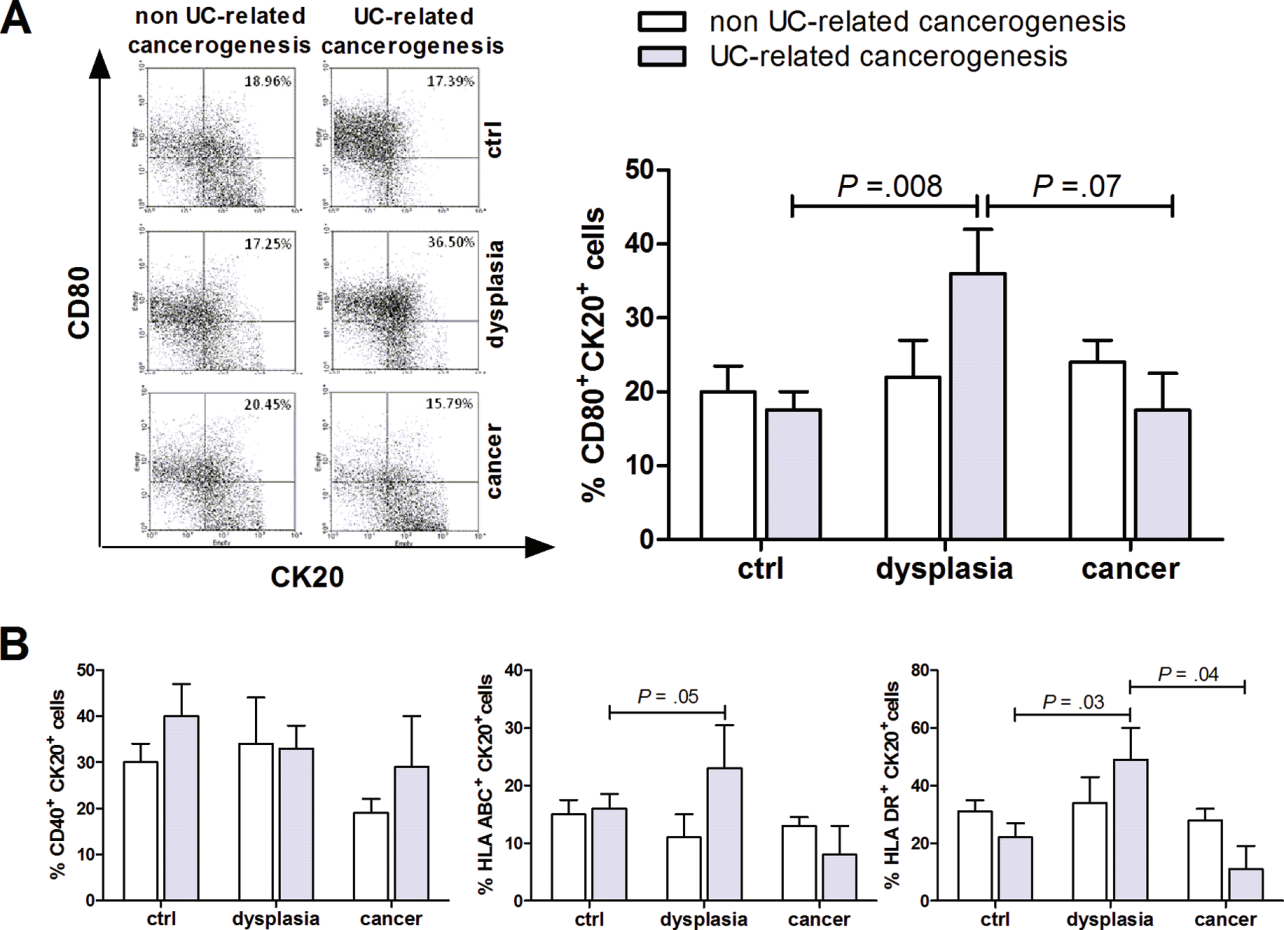
Characterization of CD80 expression on epithelial cells in human colon carcinogenesis Flow cytometric analysis of human colonic mucosa specimen showing the proportion of epithelial (Cytokeratin 20, Ck20+) cells expressing CD80 **(A)** and CD40, HLA-ABC or HLA-DR **(B)** in non UC-related and UC-related carcinogenesis. Data are presented as the mean ± S.E.M. Kruskal-Wallis test was used for comparisons.

In our series, CD80 mRNA peaked in mucosal samples from patients with UC and dysplasia and resulted down regulated in patients with UC and cancer (Supplementary Figure 1). In normal human colonic mucosa only a small proportion of cytokeratin-20-positive epithelial cells (CK20+) expresses the co-stimulatory molecule CD80, or HLA-ABC or HLA-DR (Figure 1A and 1B). However, in UC-related carcinogenesis, the percentage of CK20+ expressing either CD80+, HLA-ABC+ or HLA-DR+ increased and peaked in the dysplastic colonic mucosa (Figure 1A and 1B). On the other hand, CD40 expression remained substantially unvaried along the carcinogenic pathway (Figure 1B). Furthermore, the rate of CD80+ epithelial cells was significantly higher in UC patients with dysplasia compared with non UC-related dysplasia (Figure 1A).

### T-cell activation in human colonic carcinogenesis

**Figure 2:**
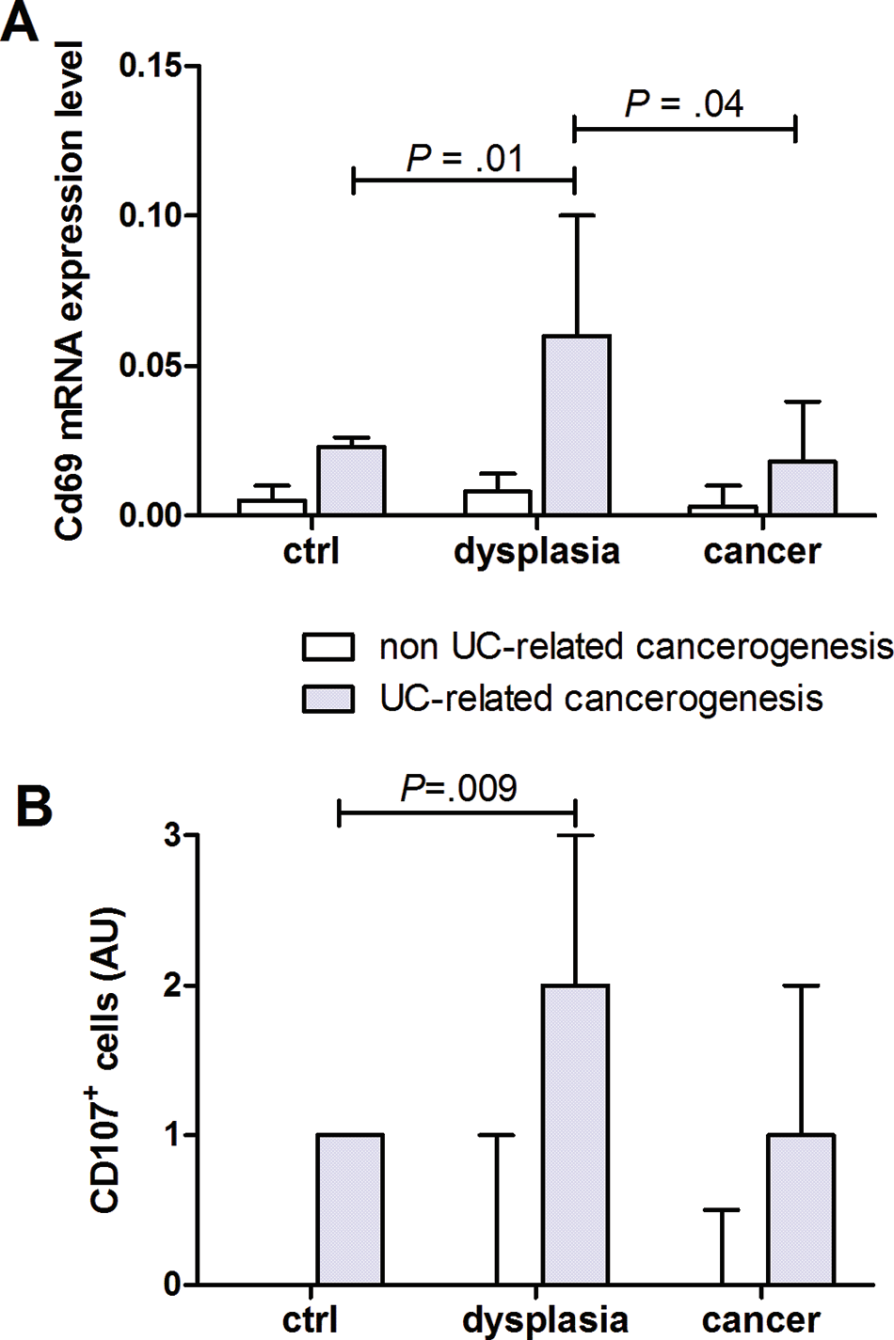
T cells activation in human colon carcinogenesis Quantification of **(A)** CD69 mRNA and **(B)** immunohistochemical quantification of CD107+ cells in human colonic mucosa specimen at different stages of non UC-related and UC-related carcinogenesis. Data are presented as the mean ± S.E.M. Kruskal-Wallis test was used for comparisons.

To determine the level of T cells activation in human colonic carcinogenesis, qRT-PCR and immunohistochemistry were used to quantitate CD69 (pan T cells activation marker) and CD107 (marker of actively degranulating cells). CD69 mRNA was significantly higher in patients with UC and dysplasia than in UC or UC and cancer (Figure 2A). Moreover, infiltration of CD107+ cells was higher in UC patients with dysplasia than in UC patients or UC with cancer (Figure 2B).

**Figure 3:**
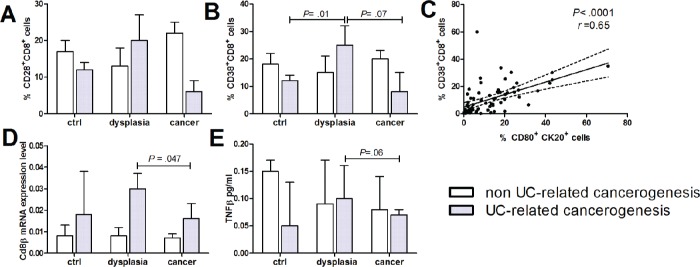
CD8+ lymphocytes infiltration and activation in human colon carcinogenesis Human intestinal mucosa specimen of non UC-related and UC-related carcinogenesis were tested for flow cytometric quantification of **(A)** CD28+ and **(B)** CD38+ CD8+ cells. **(C)** Correlation between CD80+ human intestinal epithelial cells and activated CD8 lymphocytes. **(D)** CD8β mRNA levels by RT-qPCR, **(E)** TNFβ protein levels. Data are presented as the mean ± S.E.M. Kruskal-Wallis test was used for comparisons.

Next, we characterized the cytotoxic T lymphocytes subgroup along the steps of human colon carcinogenesis. CD8+ T-cells were activated in presence of dysplastic colonic mucosa in UC patients, as demonstrated by the enhanced proportion of CD8+ lymphocytes expressing the early activation marker CD38 (Figure 3B), the high affinity CD8 receptor CD8β and the cytotoxin TNFβ (Figure 3D and 3E). Intriguingly, CD8+ lymphocytes expressing CD28, the CD80 receptor, were reduced in UC patients who developed colon cancer as opposed to patients with UC and dysplasia (Figure 3A). The synchronous presence of CD80+ epithelial cells and activated CD8+ T-cells (rho = 0.65, *p* = 0.001) suggests that the immune surveillance mechanism in UC may be mediated by the direct interaction between epithelial cells and infiltrating CD8+ T-cells (Figure 3C).

By contrast with the CD8+ lymphocytes activation described above, CD4+ lymphocytes infiltrating the colonic mucosa did not show sign of activation along the steps of UC-related carcinogenesis (Supplementary Figure 3). Indeed, T-bet, a T-box transcription factor expressed in CD4+ T lymphocytes committed to Th1 phenotype, and IL-12, a stimulator of naive T cells differentiation into Th1 cells, did not show any significant alteration along UC-related carcinogenesis pathways (Supplementary Figure 3A and 3C). Both these markers resulted higher in the adenoma than in the non UC-related cancer. IL-17, a marker of activity of Th17 subpopulation, resulted unvaried along the steps of UC-related carcinogenesis while its expression tended to be higher in the adenoma than in the healthy mucosa (Supplementary Figure 3B). Finally, IL-35, a Treg marker of activity, did not show any variation along UC-related carcinogenesis (Supplementary Figure 3D).

### *In vitro* CD8 T-cell activation is mediated by CD80-CD28 signaling

**Figure 4:**
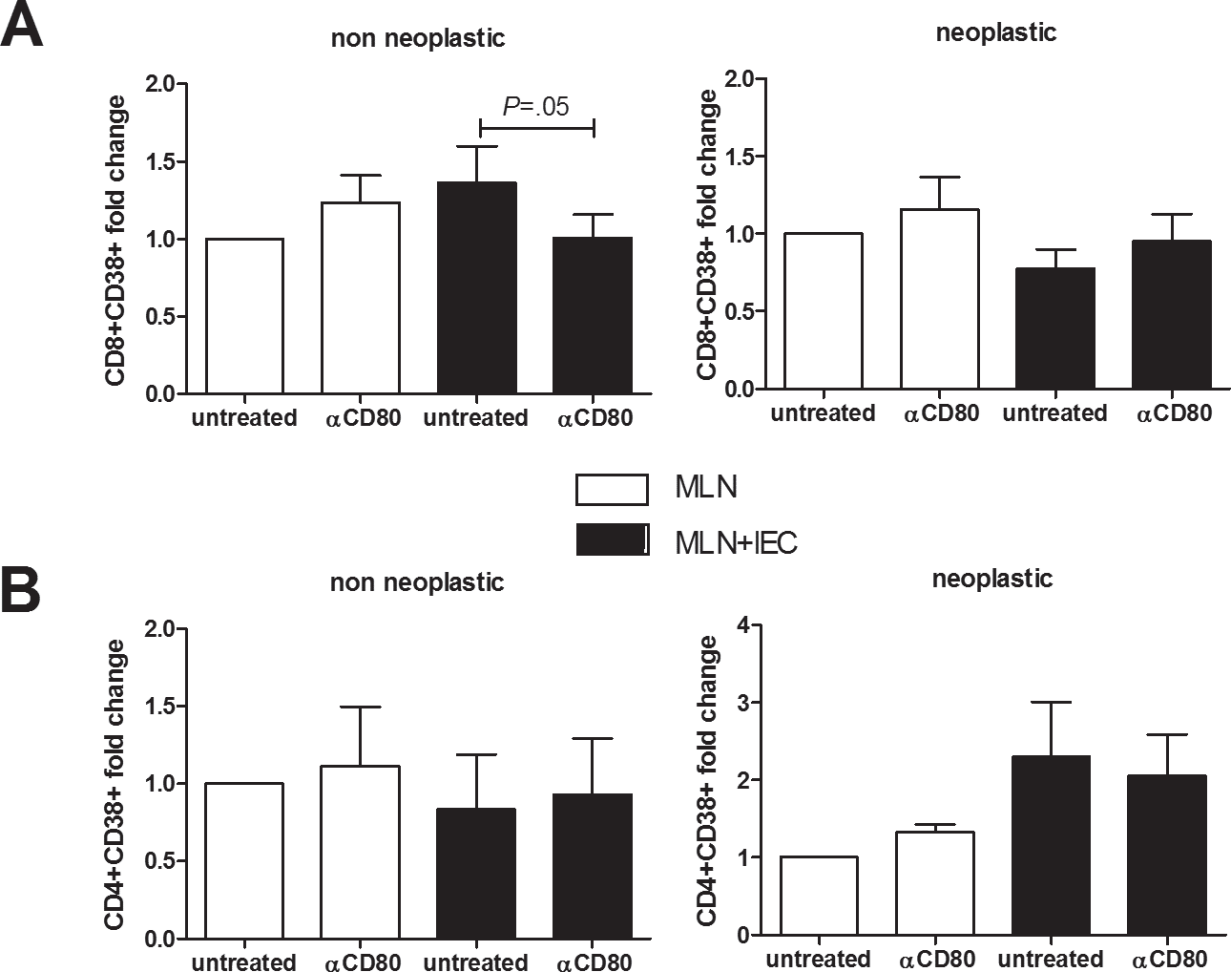
Effect of *in vitro* CD80 neutralization on lymphocytes activation MLN lymphocytes were cocultured 24 hrs with human intestinal epithelial cells derived from healthy colonic mucosa of non neoplastic and neoplastic patients (*n* = 5–12). Flow cytometric quantification of CD38+CD8+ **(A)** and CD38+CD4+ **(B)** cells is shown. Data are presented as the mean ± S.E.M. The Kruskal-Wallis ANOVA followed by Wilcoxon paired test were used for comparisons.

The simultaneous presence of activated CD8+ T-cells and CD80+ epithelial cells suggested that a direct interaction between these cells might be required for an effective immune surveillance process. Thus, we evaluated whether intestinal epithelial cells isolated from healthy colonic mucosa (obtained far from active inflammation or neoplasm) could induce a CD80-dependent activation of syngeneic lymphocytes extracted from pericolonic lymph nodes. Mucosa of patients undergoing elective operations for colonic diverticular disease or Crohn's disease (non neoplastic group) and for colonic adenocarcinoma (neoplastic group) was used. As shown in Figure 4A, epithelial cells isolated from colon of patients with inflammatory disorders provoked a 40% increase in the proportion of activated CD8+ T-cells (CD38+), that was completely blocked by anti-CD80 antibody. By contrast, in case of epithelial cells derived from patients with a colonic adenocarcinoma, there was no significant CD8+ activation (Figure 4A). Furthermore, epithelial cells co-culture triggered CD4+ T cells activation independently from CD80 signaling (Figure 4B).

### CD80 expression peaks in dysplastic epithelium in a mouse model of colonic carcinogenesis

**Figure 5:**
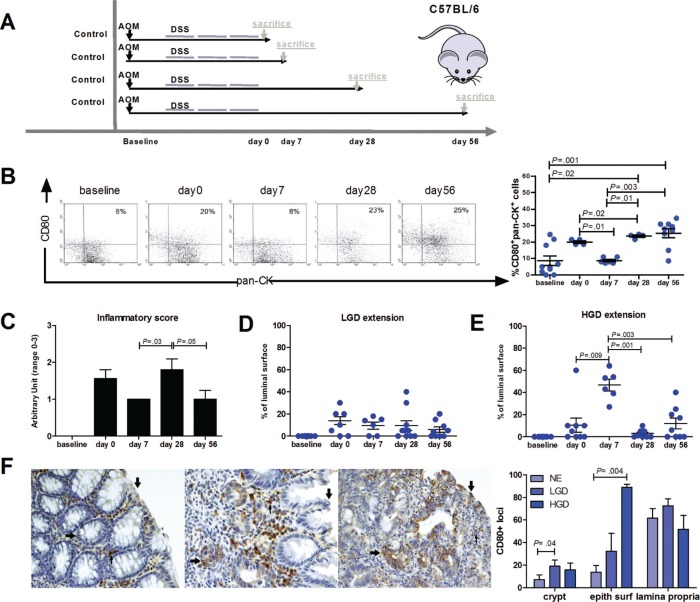
Characterization of CD80 expression and localization in a mouse model of inflammation-driven colon carcinogenesis **(A)** Scheme for the experimental course of the colon carcinogenesis model. Mice were injected with AOM i.p. at a dose of 7.4 mg/kg body weight. After 4 weeks, mice were treated with 2.5% DSS in drinking water for 5 days, followed by 2 weeks of regular water. This cycle was repeated 2 more times. Animals were sacrificed at different time points (0, 7, 28, 56 days) after the end of the last DSS cycle. **(B)** Flow cytometric analysis of CD80+ intestinal epithelial cells and quantification of **(C)** the inflammatory score, **(D)** LGD and **(E)** HGD extension during the time course of the inflammation-driven colon carcinogenesis model (*n* = 6–10 mice per group). **(F)** Representative immunohistochemical staining for CD80 of the colon from AOM/DSS treated mice exhibiting CD80+ cells in the normal epithelium, LGD and HGD areas. Arrows indicate CD80 staining on the epithelial surface (↑), colonic crypts (←) and lamina propria (↓). The frequency of CD80 positive loci present in the colonic crypts, epithelial surface and lamina propria is shown. Data are presented as mean ± S.E.M. The Kruskal-Wallis test was used for comparisons.

Next, we characterized CD80 expression in the AOM/DSS colonic inflammatory carcinogenesis mouse model (Figure 5A). Like in UC patients, there was a patchy distribution of CD80 on epithelial cells and mononucleated cells within lamina propria (Figure 5F). However, whereas CD80+ lamina propria mononucleated cells were homogeneously distributed in the presence of normal or dysplastic epithelium, CD80+ epithelial foci were significantly more frequent in the colonic crypts with LGD or HGD and in the colonic luminal surface with HGD compared with the normal epithelium (Figure 5F). In addition, we determined the time course expression of CD80 in our model. As shown in Figure 5B, the percentage of CD80+ epithelial cells showed a peak on day 0, then it exhibited a nadir at day 7, followed by another peak of expression at day 28. The inflammatory score showed a similar trend over time with a significant peak at day 28 (Figure 5C). On the contrary, HGD extension peaked at day 7 and decreased at day 28 and 56 (Figure 5E) while LGD extension was almost constant along all the steps of the experiment (Figure 5D). Notably, by day 28 approximately half of the mice spontaneously cleared HGD (Figure 5E), evidencing that in this model physiological immune surveillance is occurring.

### CD80-CD28 signaling controls murine colonic inflammatory carcinogenesis

**Figure 6:**
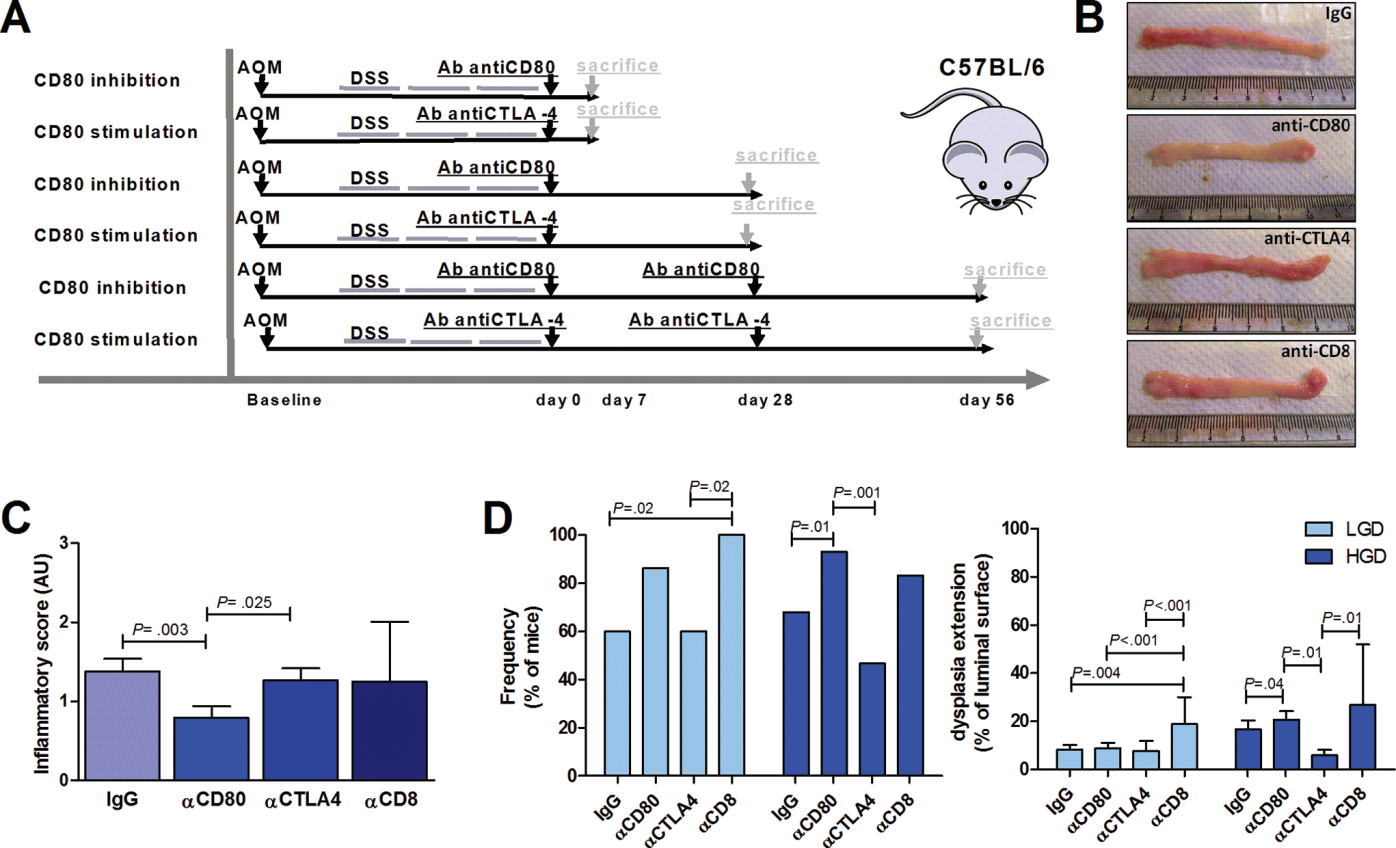
Effect of CD80 signaling modulation on the progression of an inflammation-driven colon carcinogenesis model **(A)** Scheme for the experimental course of the colon carcinogenesis model for the administration of neutralizing antibodies (200 μg/mouse) against CD80, CTLA4 or CD8. **(B)** Representative images of colons from AOM/DSS-treated mice sacrificed at day 28 after neutralizing antibodies administration. **(C)** Cumulative inflammatory scores of AOM/DSS-treated mice subjected to the administration of control IgG, anti-CD80, anti-CTLA4 or anti-CD8 antibodies (*n* = 6–25 mice per group). **(D)** Cumulative frequency and extension of LGD and HGD in AOM/DSS-treated mice subjected to administration of IgG, anti-CD80, anti-CTLA4 or anti-CD8 antibodies (*n* = 6–25 mice per group). The Kruskal-Wallis ANOVA followed by Mann-Whitney *U* test were used for comparisons.

To demonstrate the functional role of CD80 in immune surveillance during carcinogenesis progression, at the end of the AOM-DSS colitis protocol we administered neutralizing antibodies against either CD80 or CTLA4 to inhibit or enhance CD80 signaling, respectively [19] and against CD8 to assess the role of cytotoxic T cells in this process (Figure 6A–6B). In line with the role of CD80+ cells in colitis [26, 27], the inflammatory score was significantly lower in mice treated with anti-CD80 but was not affected in mice treated with anti-CTLA4 (Figure 6C). Consistent with a pivotal role for CD80 in cancer immune surveillance, blocking CD80 signaling caused a significant increase in HGD frequency and extension (*p* = 0.01 and *p* = 0.04, respectively), whereas the number and total area of HGD foci were minimal in anti-CTLA4-treated mice (Figure 6D). Moreover, blocking CD8 signaling caused an increase in both LGD and HGD extension and frequency, mimicking that caused by CD80 inhibition (Figure 6D).

### CD80-CD28 signaling shapes immune responses in experimental inflammation driven colon carcinogenesis

**Figure 7:**
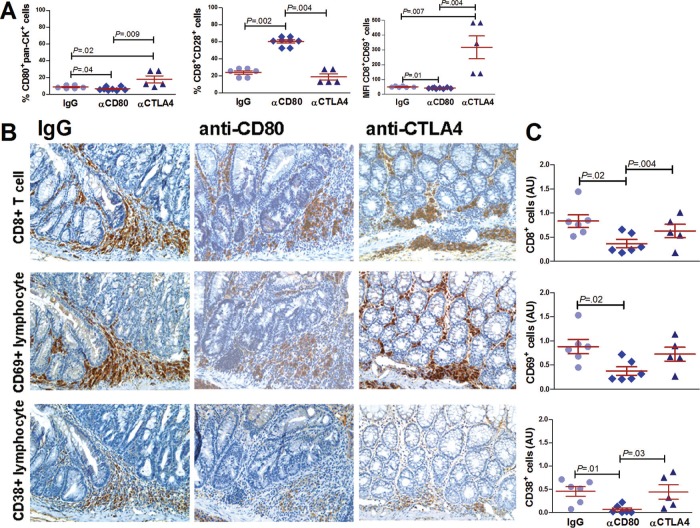
Effect of CD80 signaling modulation on CD8+ lymphocyte activation in a mouse model of inflammation-driven colon carcinogenesis **(A)** Flow cytometric analysis of CD80+ epithelial cells (pan-ck+), CD8+CD28+ and CD8+CD69+ lymphocytes from the colons of AOM/DSS-treated mice subjected to i.p. administration of 200μg of control IgG, anti-CD80 or anti-CTLA4 antibodies. Animals were sacrificed on day 7 (*n* = 5–6 mice per group). Representative immunohistochemical staining **(B)** and quantification **(C)** of CD8, CD69 and CD38 cells of the colons of AOM/DSS-treated mice subjected to the administration of control IgG, anti-CD80 or anti-CTLA4 antibodies sacrificed at day 7 (*n* = 5–6 mice per group) (40x magnification). Data are presented as the mean ± S.E.M. The Kruskal-Wallis ANOVA followed by Mann-Whitney *U* test were used for comparisons.

Because the main immune surveillance mechanisms seemed to occur at an early stage following the inflammatory insult in the AOM-DSS model of colonic inflammatory-mediated carcinogenesis, we analyzed the immunological events taking place at this time point (day 7). Anti-CD80 administration caused a significant increase in CD28+ CD8+ lymphocyte (Figure 7A). Moreover, anti-CTLA4 treatment increased the proportion of CD80+ epithelial cell and the expression of the activation marker CD69 on CD8+ T cells (Figure 7A). In fact, the massive presence of CD8+ lymphocytes in dysplastic areas suggests that these cells may be the main executors of the CD80 immune surveillance-driven process in the colon. Indeed, the number of early-activated lymphocytes (CD38+ and CD69+) is reduced in dysplastic lesions of mice treated with anti-CD80 as opposed to the anti-CTLA4-treated mice (Figure 7B and 7C).

## DISCUSSION

Formulated in the early seventies [21], the immune surveillance theory has always been difficult to demonstrate. Dunn and co-workers [22] revamped it by proposing the cancer immunoediting theory, according to which a tumour interacts with the immune system in three sequential steps: an elimination phase during which the immune system clears out most tumour cells; an equilibrium phase during which an adaptive immune response maintains occult cancer in an equilibrium state; and finally, an escape phase during which a tumour grows, invades, and metastasizes [23]. Pages and co-workers demonstrated that in non-inflammatory CRC an increase in T-cell migration, activation and differentiation ensures a sort of equilibrium phase in tumours without vascular embolism, lymphatic invasion or perineural infiltration [24]. In UC, the discrepancy between the dysplasia rate and the actual cancer incidence suggests that an elimination phase may actively prevent progression from dysplasia to cancer. We postulate that CD80 over-expression, linked to oxidative DNA damage in the colonic mucosa of UC patients with dysplasia, may play an important role during this elimination phase [18, 19, 25].

Our results suggest that colonic epithelial cells may act as non professional APCs in UC-related colonic carcinogenesis through CD80, HLA-ABC and HLA-DR upregulation. Interestingly, a recent study showed that at least 20 genes, including cancer-related ones, had a differential expression in the healthy mucosa of UC patients with cancer as compared to the mucosa of UC patients without cancer [26]. Taken together, these observations suggest that, in the early stages of neoplastic transformation, the intestinal epithelium might show a distinct antigenic profile which, in association to CD80 and HLA-ABC, may be recognised and eliminated by the immune system.

An active role in the immune surveillance mechanisms of CD8+ lymphocytes expressing CD28, the CD80 receptor essential for T cell activation [27], is supported by their diminished number at the cancer stage in patients with UC. Koch et al. demonstrated a tumor-specific activation and cytotoxic activity of CD8+ tumour infiltrating lymphocytes (TIL) against tumor antigens in early CRC and several authors had already reported a down-regulation of CD8 activation and infiltration in advanced CRC [19, 28–30]. Moreover, among patients with CRC, an apparent ‘protective’ function associated to high CD8+ lymphocyte infiltration was enhanced by nuclear expression of IL-23p19 within cancer cells [31]. Our data indicate that UC-related precancerous lesions are an even more effective stimulus to activate CD8+ lymphocytes. Moreover, the synchronous presence of CD80+ epithelial cells and activated CD8+ T-cells suggests that the immune surveillance mechanism in UC may be mediated by the direct interaction between epithelial cells and mucosal CD8+ T-cells. Indeed, Hodge et al. demonstrated that tumor cells transduced with CD80 gene by means of a retroviral vector became susceptible to T cell-mediated elimination [32].

To show the ability of IEC to directly modulate lymphocyte activation through CD80 signaling, we co-cultured IEC from healthy colonic mucosa with syngeneic lymphocytes extracted from pericolonic lymph nodes of patients undergoing surgery for non neoplastic disease and for colonic adenocarcinoma. A limitation of this assay was that we used IEC from patients with Crohn's disease and diverticulitis in remission instead of those from UC patients. Indeed, we deliberately used them because non inflamed colonic tissue can be much easily retrieved from surgical specimen of non UC-patients. IEC isolated from patients with non neoplastic disease provoked an increase in the proportion of activated CD8+CD38+ T-cell that was significantly blocked by the addition of anti-CD80 antibody, whereas no significant changes were observed in terms of CD4 activation. By contrast, IEC isolated from neoplastic colon failed to activate CD8+ T-cells, supporting the view that APC function or CD8+ T-cell function (or both) are inhibited in presence of invasive cancer [33]. This is consistent with a previous study showing that stimulation with mucosal homogenate from intestinal segments containing dysplastic epithelium resulted in vigorous proliferation of T lymphocytes from mesenteric nodes draining the dysplastic-colonic region [33].

To begin to dissect the *in vivo* functional role of CD80 in colonic inflammatory carcinogenesis, we used AOM-DSS colitis as a mouse model of inflammatory colonic carcinogenesis [34, 35]. The prevalence of CD80+ epithelial foci in correspondence of LGD or HGD, as opposed to the homogeneously distributed CD80+ lamina propria mononucleated cells in the presence of normal or dysplastic epithelium, further suggests that epithelial cells, in addition to professional APCs, are involved in the cross-talk with T lymphocytes in colon carcinogenesis. Interestingly, the number of CD80+ epithelial cells followed a trend similar to that of the inflammatory score over time, whereas an opposite trend between CD80 expression and HGD extension was observed, thus evidencing the possibility that either inflammation and tumorigenic stimuli contribute to the regulation of CD80 expression in this experimental model with distinct chronological patterns. Consistent with a pivotal role for CD80 in cancer immune surveillance, neutralization of CD80 caused a significant increase in HGD, whereas enhancing CD80 signaling with anti-CTLA4 protected mice from colon carcinogenesis progression. Together, these data confirm that CD80 signaling plays a prominent role in the immune surveillance mechanisms, as CD80 impairment enables the progression from LGD to HGD without affecting the appearance of LGD foci. In our opinion, in HGD, but not LGD, antigens that epithelial cells can present in association with CD80 are specifically expressed. However, CD80 signaling augmentation by means of anti-CTLA4 administration failed to completely eradicate the dysplasia. This might be explained by the AOM-DSS carcinogenesis model mechanism through random DNA damage that might involve CD80 gene regulation or the regulation of other genes involved in the CD80 signal cascade.

The analysis of the immunological events occurring early after the inflammatory insult in our model of CRC revealed that CD80-CD28 signaling pathway blockade results in inadequate lymphocytes activation that might induce antigen-specific tolerance and tumor progression [36]. By contrast, anti-CTLA4 treatment increased the proportion of CD80+ epithelial cell and the expression of CD69 on CD8+ T cells. Indeed, the presence of activated CD8+ lymphocytes in dysplastic areas suggests that these cells may be the main executors of the CD80 immune surveillance process in the colon. In line with this view, CD8 neutralization caused a significant increase in HGD extension and frequency similarly to CD80 inhibition, proving that cytotoxic lymphocytes can control the progression of the early stages of inflammatory colonic carcinogenesis.

Taken together, our data support the hypothesis that in colonic inflammatory carcinogenesis the progression from dysplasia to invasive cancer is controlled not by a mere immunoediting process, such as that observed in sporadic invasive cancer [30], but by a truly effective immune surveillance mechanism mediated by CD80 expression on epithelial cells that can completely clear preneoplastic lesions in a large proportion of cases. The dysregulation of this immune checkpoint may lead to the progression from LGD to HGD and invasive cancer in UC [29]. Thus, blocking antibodies against CTLA4 such as ipilimumab, already approved for clinical use in melanoma patients to down regulate tumor-induced tolerance and immune suppression [37], might find a clinical application in colonic carcinogenesis [38]. To our knowledge, only one phase II trial with anti-CTLA4 monoclonal antibody has been conducted on advanced CRC patients [39]. The lack of significant clinical activity reported in this trial may be explained by the fact that only one patient (out of 47) received more than one dose of antibody. Although a relatively poor-prognosis group of heavily pretreated patients was recruited, 43% of these patients were still alive at 6 months [39]. Moreover, in studies on advanced melanoma patients ipilimumab did not change median survival but, in the subgroup of patients who responded, it significantly prolonged survival over any expectation [37].

In conclusion, this study provide new insights about the mechanisms of immune surveillance in UC-associated colon carcinogenesis, thus having a potential impact in the early risk prediction and prevention of colon cancer. Future clinical studies will be required to evaluate the potential value of the use of immunotherapy in combination with other conventional treatment approaches to prevent, control, and/or eradicate established colon cancer.

## MATERIALS AND METHODS

### Patients

A prospective cohort of 153 patients who completed a colonoscopy or underwent colonic resection for either UC (with or without colonic dysplasia or colon cancer), colonic adenoma or CRC was enrolled. Furthermore, 47 healthy subjects who underwent colonoscopy for colonic cancer screening were recruited as controls. Mucosal samples were obtained from colonic biopsies of normal (sigmoid colon), neoplastic or preneoplastic mucosa (macroscopic lesion). Diagnosis was confirmed by clinical, radiological and histological parameters. The UC patients were receiving 5-ASA or steroids; none of the patients were treated with azathioprine, cyclosporine or anti-TNFα within the last six months. Moreover, patients treated with chemo- or radiotherapy in the previous six months were excluded. The study was performed according to the principles of the Declaration of Helsinki, all participants provided informed consent, and IRB approval (Veneto Institute of Oncology, Padova, Italy) was obtained. The characteristics of the patients and controls are outlined in Supplementary Table 1.

### Isolation of human intestinal epithelial cells (IEC) and mesenteric lymph node (MLN) lymphocytes

Human IEC and MLN lymphocytes were isolated from normal colonic tissue and mesenteric lymph nodes, respectively, obtained from patients undergoing surgery for CRC, ileal Crohn's disease or diverticulitis. IEC were isolated and cultured as previously reported [40]. See Supplementary Methods for details.

### IEC:MLN lymphocytes co-culture assay

Human MLN lymphocytes were suspended at 1 × 10^6^ cells/ml in DMEM with 10% FBS, 2.5% Penicillin-Streptomycin-Fungizone and 1% Gentamicin and cultured either alone or with syngeneic primary human IEC for 24 hours, in the presence or absence of neutralizing antibodies against CD80 (eBioscience). After the culture period, lymphocytes were harvested by vigorous pipetting and washed three times with PBS. Cells were then stained for CD4, CD8 and CD38 and analyzed by flow cytometry.

### Mouse colon cancer model

Animal experiments were performed according to Italian Law 116/92 and European directive 2010/63/UE. Experimental protocols were reviewed and approved by the Institutional Animal Care and Use Committee (“Comitato Etico Scientifico per la Sperimentazione Animale”) of the University of Padova, Padova, Italy. Mice were maintained under standard laboratory conditions with 12:12-h light-dark cycles and free access to regular rodent chow food and water at all stages of the experimental model. We obtained male 8-week-old C57Bl6/J mice from Harlan Laboratories S.r.l. Cohoused 10-wk-old mice were injected with azoxymethane (AOM, Sigma) i.p. at a dose of 7.4 mg/kg body weight (mean weight 27 ± 1gr). After 4 weeks, mice received 2.5% dextran sodium sulphate (DSS, Applichem) (M.W. 40,000 g/mol) in the drinking water for 5 days, followed by 2 weeks of regular water. This cycle was repeated two more times [34].

Mice were randomized into four groups and treated i.p. with 200 μg/mouse of either control IgG, anti-CD80 Ab (clone 16-10A1, ATCC hybridoma no. HB-301), anti-CTLA4 Ab (clone UC10-4F10-11, ATCC hybridoma no. HB-304) or anti CD8 (clone 2.43, ATCC hybridoma no. TIB-210) monthly, starting one week after the third DSS treatment [35, 41]. All mice were housed in the same animal room and sacrificed at different time points (1 week, 4 weeks and 8 weeks) after the first antibody injection. Colons were removed, flushed with PBS and a segment of the most distal colon above the anus was collected and processed for flow cytometric analysis. Remaining tissue was fixed as “Swiss rolls” in 10% neutral-buffered formalin and paraffin embedded for histology.

### Histopathology

Sections (3 μm) from formalin-fixed and paraffin-embedded human and mice specimens were stained with hematoxylin-eosin. Histological inflammation was quantified and classified by a pathologist (S.M.) unaware of the arm of the experiment using Floren's score [42] and the Vienna classification of gastrointestinal epithelial neoplasia [43]. The classification of epithelial neoplasia was supported by Ki67 staining (Supplementary Figure 2). Murine colons were analyzed for dysplasia at high magnification (40x). The extent of dysplasia was quantified as the percentage of involved bowel length. Inflammation was scored from 0 to 4, with normal colonic mucosa = 0, shortening and loss of the basal one-third of the crypts with mild inflammation in the mucosa = 1, loss of the basal two-thirds of the crypts with moderate inflammation in the mucosa = 2, loss of the entire crypts but not the surface epithelium with severe inflammation in the mucosa = 3, and loss of both entire crypts and surface epithelium with severe inflammation in the mucosa = 4 [44].

### Immunohistochemistry

Immunohistochemical analyses were performed using standard procedures, and the resulting sections were evaluated by a single pathologist in a blinded fashion. CD80 expression was graded on a semi-quantitative scale (negative, low, moderate or high). The frequency of CD80+ cells was assessed by counting the number of positive cells in a hundred cells in a representative field (LGD, HGD and normal epithelium). Ten random fields (40x) from each sample were examined. The expression of CD8, CD69 and CD38 was measured semi-quantitatively (none, mild, moderate and severe infiltration). The number of fields (40x) with moderate/severe infiltration per intestinal surface was obtained and considered a parameter of the immune response to early epithelial mutations. The antibodies used are summarized in Supplementary Table 2.

### RNA extraction and qRT-PCR

Total RNA from intestinal tissue was extracted using the SV Total RNA Isolation System (Promega) according to the manufacturer's instructions. Complementary DNA (cDNA) synthesis was performed using the Applied Biosystems cDNA Synthesis kit according to the manufacturer's directions. Specific mRNA transcripts were quantified with SYBR Green PCR Master Mix in an ABI PRISM 7000 Sequence Detection System (Applied Biosystems). The expression of the target molecule was normalized to the expression of the Actab housekeeping gene. The primers used are summarized in Supplementary material.

### Immunoassay

Tissues were mechanically homogenized in 500 μl of PBS (1:10 wt/vol ratio) containing a protease inhibitor cocktail (Roche Applied Science). Protein concentration was determined using the BCA Protein Assay kit (Pierce). Mucosal levels of IL-12, TNF-β, granzyme B (Bender MedSystems GmbH), IL-17 (R&D Systems), T-bet (CUSABIO) and IL-35 (Uscn Life Science Inc) were measured by immunometric assay according to manufacturer's instructions. Data are expressed in pg per μg of total proteins.

### Flow cytometry

Human intestinal mucosa was stripped from the muscularis mucosa, cut into strips, and freed of mucus by a 30-min wash in HBSS containing 10 mM DTT (Applichem). IEC were isolated by 30-min incubation of the intestinal mucosa in HBSS containing 1 mM EDTA (Sigma-Aldrich). For LPMC, stripped mucosa, freed of mucus and IEC, was digested with 1 mg/ml collagenase and DNase (Sigma Aldrich) for 30 min at 37°C. The resulting crude cell suspensions were purified using a Ficoll-Hypaque Plus gradient (GE Healthcare), and the preparations were preferentially enriched for LPMC, washed, and collected. Mice colonic samples were freed of mucus by a 30-min wash in HBSS containing 10 mM DTT (Applichem). Then the mucosa was removed by the underlying muscle layer, and digested with 1 mg/ml collagenase and DNase (Sigma Aldrich) for 30 min at 37°C. Preparations were then enriched preferentially for LPMC using a Ficoll-Hypaque Plus gradient (GE Healthcare).

For staining of freshly isolated cells, 105 cells were stained in PBS/2% FBS with appropriate combinations of FITC- and PE-conjugated antibodies for 30 min on ice. Single-cell suspensions were subjected to flow cytometry to determine the proportion of epithelial cells (Cytokeratin 20, Cyt-20+) acting as antigen-presenting cells (expressing CD80, CD40, HLA ABC or HLA DR) and the proportion of activated CD8+ T cells (positive for CD28, CD38 or CD69). Flow cytometric analysis was performed using a FACSCalibur based on CellQuest software (Becton Dickinson). The antibodies used are summarized in Supplementary Table 2.

### Statistics

Data are presented as the mean +/− SEM. The non-parametric Mann–Whitney's *U*-test for independent variables, the Wilcoxon test for paired matched variables or Kruskall-Wallis ANOVA for multiple variables were used for comparisons as appropriate. The Kendall rank correlation test was performed. Differences were considered significant at *P* < 0.05.

## SUPPLEMENTARY METHODS FIGURES AND TABLES


