# Supplementary material for: CD80-CD28 signaling controls the progression of inflammatory colorectal carcinogenesis
Source: Oncotarget. 2015 Jan 16;6(24):20058–69. doi: 10.18632/oncotarget.2780 (PMC4652987; doi:10.18632/oncotarget.2780)
Supplement: Supplementary file 1 [file oncotarget-06-20058-s001.pdf]

SUPPLEMENTARY METHODS

ISOLATION OF HUMAN INTESTINAL EPITHELIAL CELLS (IEC) AND MESENTERIC LYMPH NODE (MLN) LYMPHOCYTES

Briefly, following dissection of the mucosa into small strips and mucus removal by 1 mM DTT (Applichem) in HBSS 30 min at room temperature, mucosal strips were incubated in 1 mM EDTA for 10 min at 37°C. Mucosal strips were briefly rinsed in HBSS and transferred to fresh HBSS followed by 10 vigorous shakes of the container. This procedure leads to the instant detachment of IEC in a full-length crypt formation. Rapid purification of detached IEC was achieved using a mesh filter (80- µm pore size, Sigma); the suspension containing the IEC crypts was passed over the mesh, separating ("harvesting") the IEC crypts in the filter from single cells. The filter was inverted, and purified intact IEC crypts were washed out with culture medium (DMEM with 10% heat inactivated Fetal Bovine Serum (FBS), 2.5% penicillin-streptomycin-Fungizone and 1% gentamicin, all from Gibco). The IEC crypts solution was then transferred to a collagen I-coated (20 µg/cm^2, Sigma) 96-well plate for seeding of the cells. After 3 hours, non-attached, apoptotic IEC were removed, and fresh culture medium was added. For human MLN lymphocytes, the lymph nodes were cut finely with scissors and then gently pressed through a sterile stainless steel mesh (80-µm pore size, Sigma) to obtain a single-cell suspension. Mononuclear cells were enriched by Ficoll-Paque PLUS (GE Healthcare) gradient for 20 minutes at 600 x*g* at room temperature. Monocytes were excluded from the preparation by adherence to tissue culture plates for 1 hour. MLN lymphocytes were collected, washed, counted and prepared for the subsequent assay.

RNA extraction and qRT-PCR

The primers used were:

- human CD80: FW 5′-CTCACTTCTGTTCAGGTGT-TATCCA-3′; RV 5′-TCCTTTTGCCAGTAGAGC-GA-3′;
- human CD3γ: FW 5′-GGGATGTATCAGTGTA-AAGG-3′; RV 5′-CAGCAATGAATAGACCC-3′;
- human CD8b: FW 5′-TCATTCTCAATCTCCAAGC-GTG-3′; RV 5′-GAAGGAAATCAACCACACT-CAGC-3′;
- human CD69: FW 5′-CAAGTTCCTGTCCTGTGT-GCTG-3′; RV 5′-GCCCACTGATAAGGCAAGAG-3′;
- human Actin: FW 5′-CTGGACTTCGAGCAAGA-GATG-3′; RV 5′-AGTTGAAGGTAGTTTCGTG-GATG-3′.

SUPPLEMENTARY FIGURES AND TABLES

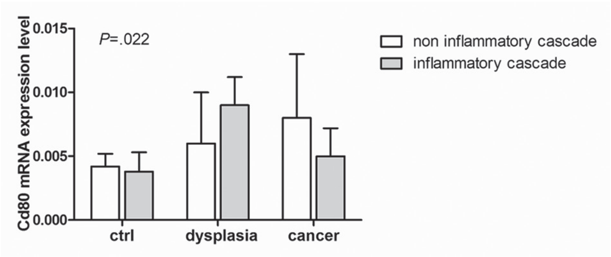

Supplementary Figure 1: Quantification of CD80 mRNA in the colonic mucosa at different stages of non UC-related and UC-related carcinogenesis. Data are presented as the mean ± S.E.M. Kruskal-Wallis test was used for comparisons.

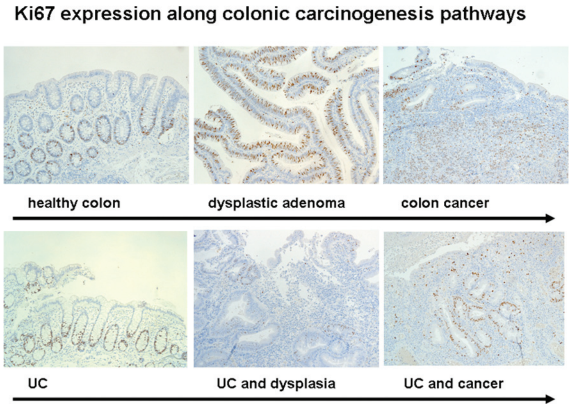

Supplementary Figure 2: Representative Ki67 staining of colonic mucosa at the different stage of non UC-related and UC-related carcinogenesis.

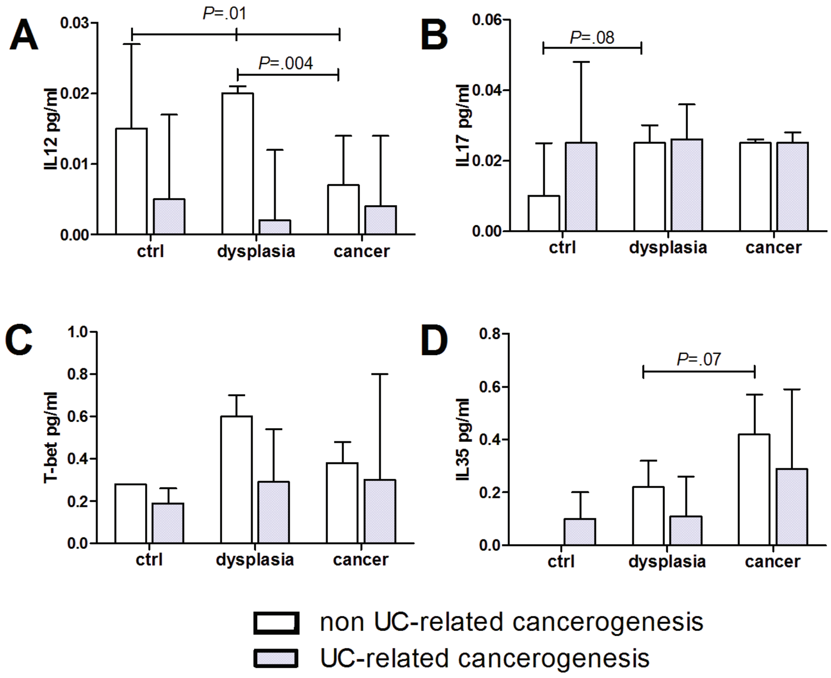

Supplementary Figure 3: Human colonic mucosa specimen of non-UC related and UC-related carcinogenesis were tested for protein levels of IL12 (A), IL-17 (B), T-bet (C) and IL35 (D) by ELISA. Data are presented as the mean ± S.E.M. Kruskal-Wallis test was used for comparisons.

Supplementary Table 1: Patients' characteristics

Groups	Healthy subjects	Adenoma with dysplasia	Invasive CRC	UC	UC and dysplasia	UC and CRC
Total patients	47	32	18	73	19	11
Female / Male	26 / 21	15 / 17	7 / 11	26 / 47	10 / 9	5 / 6
Median age (IQR)	62 (52.25–72.5)	66 (55–73.25)	67 (59.25–77.25)	48 (41.25–60)	58 (46–69.5)	56 (49–63.25)
Colonic inflammation	Floren severity score					
0	47	32	18	17	5	0
1	0	0	0	13	1	4
2	0	0	0	11	4	3
3	0	0	0	32	9	4
HGD	NA	16	3	NA	5	3
LGD	NA	30	7	NA	14	4
TIS			1			3
T1N0M0			1			3
T2N0M0			4			0
T2N1M0			2			0
T3N0M0			5			1
T3N0M1			1			0
T3N1M0			0			2
T3N1M1			2			1
T3N2M0			1			0
T3N2M1			1			0
T4N2M1			1			1
History of CRC	9	7	0	0	0	4
History of colonic dysplasia	0	11	2	11	9	7
History of CT/RT	4	2	0	0	0	0
Familial history of CRC	4	9	3	7	1	0

HGD, high-grade dysplasia; LGD, low-grade dysplasia; RPC, restorative proctocolectomy

Supplementary Table 2: Antibodies used in the study

Antibody	Application	Clone	Source
Anti-human CD8a PE	Flow cytometry	HIT8a	eBioscience Inc., San Diego, CA
Anti-human CD28 FITC	Flow cytometry	CD28.2	eBioscience Inc., San Diego, CA
Anti-human CD38 FITC	Flow cytometry	HIT2	eBioscience Inc., San Diego, CA
Anti-human CD40 FITC	Flow cytometry	5C3	eBioscience Inc., San Diego, CA
Anti-human CD80 FITC	Flow cytometry	2D10	eBioscience Inc., San Diego, CA
Anti-human CD80	neutralization	2D10.4	eBioscience Inc., San Diego, CA
Anti-human HLA-DR FITC	Flow cytometry	LN3	eBioscience Inc., San Diego, CA
Anti-human HLA-ABC FITC	Flow cytometry	W6/32	eBioscience Inc., San Diego, CA
Anti-human cytokeratin 20	Flow cytometry	Ks20.8	Abcam Ltd.,UK Cambridge
Anti-mouse IgG TRITC	Flow cytometry		Zymed, Vienna, Austria
Anti-mouse CD8a (Ly-2) PE-Cy7	Flow cytometry	53–6.7	eBioscience Inc., San Diego, CA
Anti-mouse CD28 FITC	Flow cytometry	E18	Abcam Ltd., UK Cambridge
Anti-mouse CD38 FITC	Flow cytometry	90	eBioscience Inc., San Diego, CA
Anti-mouse CD69 PE	Flow cytometry	H1.2F3	Abcam Ltd., UK Cambridge
Anti-mouse CD8	IHC	GWB-BEBE35	GenWay Biotech, Inc., San Diego, CA
Anti-mouse CD38	IHC	50191-R032	Sino Biologicals Inc.,, Beijing,, China
Anti-mouse CD69	IHC	ab130185	Abcam Ltd., UK Cambridge
Anti-mouse CD80	IHC	EP1155Y	Abcam Ltd., UK Cambridge
Anti-mouse CD80 FITC	Flow cytometry	16-10A1	eBioscience Inc., San Diego, CA
Anti-mouse pan Cytokeratin PE	Flow cytometry	C-11	Abcam Ltd., UK Cambridge
